# Associations between violent crime inside and outside, air temperature, urban heat island magnitude and urban green space

**DOI:** 10.1007/s00484-023-02613-1

**Published:** 2024-01-08

**Authors:** Heather R. Stevens, Petra L. Graham, Paul J. Beggs, Alessandro Ossola

**Affiliations:** 1 School of Natural Sciences, Faculty of Science and Engineering, Macquarie University, Macquarie Park, New South Wales 2109 Australia; 2 School of Mathematical and Physical Sciences, Faculty of Science and Engineering, Macquarie University, Macquarie Park, New South Wales 2109 Australia; 3 Department of Plant Sciences, University of California Davis, Davis, CA 95616 USA

**Keywords:** Crime, Urban microclimate, Public health, Green space, Urban greening, Urban forests, Heat stress

## Abstract

There are more incidents of violence in summer and on hot days, a trend likely to be exacerbated by climate change. Urban areas experience additional temperature modulation due to the urban form, however, to date, no studies have considered the effect of the urban heat island (UHI) or green space with respect to the temperature–violence relationship. This study modelled the relationship between the number of daily violent crime incidents that occurred inside or outside between July 2013 and June 2018, and the average surface UHI or percentage greencover (including grasses, shrubs and trees) within each local government area in Greater Sydney, Australia. Panelised negative binomial time series regression models indicated that the violent crime rate was associated with higher surface UHI for crimes committed outside (*p* = 0.006) but not inside (*p* = 0.072). Greater percentage of all vegetation was associated with significantly lower rates of violent crime committed outside (*p* = 0.011) but was not associated with violent crimes committed inside (*p* = 0.430). More socio-economic disadvantage was associated with higher rates of violent crime committed inside (*p* = 0.002) but not outside (*p* = 0.145). Greater temperature was non-linearly associated with higher rates of violent crime committed both inside and outside (*p* < 0.001). The findings of this study are important because both violence and heat exposure are critical health issues and will be stressed by urbanisation and climate change. The expansion of green space and/or reduction in UHI may mitigate these effects.

**Supplementary Information:**

The online version contains supplementary material available at 10.1007/s00484-023-02613-1.

## Introduction

Violent crime is generally higher in summer and increases with temperature (Anderson 2001, 2012; Burke et al. 2015; Hsiang & Burke 2014; Lynch et al. 2020). Empirical studies on temperature-related violence often investigate aggregated counts of assault temporally, including hourly and daily (Butke & Sheridan 2010; Linning et al. 2017; Stevens et al. 2021), heatwave events (Xu et al. 2020, 2021), seasons (Stevens et al. 2019) and even decadal/generational trends (Van de Vliert 2008). Some studies also compare temperature-related violence across *space*, like indoor versus outdoor (Rotton & Cohn 2004), and within and between cities and countries (Brunsdon et al. 2009; Lester 1986; Mares & Moffett 2016).

The causal mechanisms driving temperature-related violence are heterogeneous and interconnected; however, generally temperature has an effect on person and/or situation (Allen & Anderson 2017; Felson 2016). It has been long proposed that in warmer temperatures, people engage in more socialisation which fosters motivations and opportunities to act aggressively (Rotton & Cohn 2003). In extreme heat, people may experience physiological stress like lethargy or retreat from the heat by sheltering indoors (Bell 1992; Obradovich et al. 2017), and this physical and/or social stress can also provoke aggressive behaviours. Higher alcohol consumption, both in social settings and in the home, is also associated with warmer weather (Hagström et al. 2019) and increases in violent crime (Descallar et al. 2012).

### Urban heat island, greening and violent crime

Almost all temperature-related violence studies have used ambient air temperature to represent heat exposure; Huang et al. (2011) is the only study that has considered the effect of land surface temperatures on crime (finding a positive association). No studies to date have specifically considered the association between crime and the urban heat island (UHI) effect, that is, the additional heat in the built-up environment from hard surfaces, which absorb, store and radiate heat (Oke 1982). The surface temperature within a city varies more than air temperature (Chakraborty et al. 2022). Cities like Sydney, Australia, already experience elevated temperatures from the UHI (Santamouris et al. 2018), and the effect is increasing with population growth and urban densification (Levermore et al. 2018).

Urban greencover (including forests, parks, gardens, street trees and green roofs) (Markevych et al. 2017) is associated with lower land surface temperatures compared to non-vegetated surfaces (Maimaitiyiming et al. 2014). This can mitigate the effects of heatwaves (Schubert & Grossman-Clarke 2013; Ossola et al. 2021) and reduce the UHI effect (Maimaitiyiming et al. 2014; Tran et al. 2006). In fact, vegetation can both absorb and reflect solar radiation better than non-vegetated surfaces and has a cooling effect through evapotranspiration (Maimaitiyiming et al. 2014; Salmond et al. 2016). Tall vegetation like trees can provide shade to pedestrians, as well as buildings and parked cars, avoiding thermal accumulation inside (Bonan et al. 2002; Mavrogianni et al. 2014). The cooling effect of greencover is dependent on the type (size, leaf width, etc.), density, placement and irrigation (Grimmond & Oke 1999; Ossola et al. 2021; Wilmers 1990). Greencover has the potential to reduce the effects of climate change through mitigation like reduced energy use from less air-conditioning and carbon sequestration, and adaptation like evaporative cooling and increased urban albedo (Sun et al. 2019; Cheela et al. 2021).

Increased greencover is also generally associated with less crime. Shepley et al. (2019) reviewed 45 US studies on violent crime and greencover, finding that, while studies used varying greencover types (i.e. street trees, parks), sizes (small gardens to large parkways), crimes (i.e. shootings, assaults) and methodologies, most found that violence decreased as greencover exposure increased. Studies have also looked at the change over time in crime rates from remediating land. In a meta-analysis on greening vacant lots and impacts on firearm violence, it was found that greening and gardening interventions reduced firearm violence by around 5.5%; however, just mowing the grass did not show a reduction in gun crime (Sadatsafavi et al. 2022). Similarly, greening vacant land has also shown to improve mental health (South et al. 2018).

### The disproportionate effect of temperature-related violence among communities

Crime rates, access to green space and heat exposure are disproportionate within the population. For example, in Australia, those living in an area with higher socio-economic disadvantage (i.e. low socio-economic status (SES) areas) are more than twice as likely to experience physical assault than those in more affluent areas (Australian Institute of Health and Welfare 2018), a trend that is also found elsewhere (Chakraborty et al. 2019; Lockwood 2007). Poorer neighbourhoods also generally have lower urban greening (Astell-Burt et al. 2014; Li & Liu 2016; Wolch et al. 2014). Furthermore, those in low SES areas are more likely to experience heat exposure; needing to work outdoors (Carey et al. 2014), walk for transport, or having limited access to private transportation (Goldsworthy & Poruschi 2019; Turrell et al. 2013). Low SES areas may also have higher density of alcohol outlets (Livingston 2012). These factors may explain, in part, why temperature-related violence is disproportionately higher for those more disadvantaged (Coccia 2017; Harries et al. 1984; Otrachshenko et al. 2021).

The studies described above have investigated the associations between temperature and violence, green space and violence, and green space and temperature. However, no studies to date have simultaneously considered the association between violent crime and UHI or temperature and green space. This study addresses that gap by investigating the associations between violent crime and maximum temperature together with either surface UHI, or percentage of different types of greencover (grass, shrub and tree or all vegetation) while adjusting for socio-economic disadvantage. The findings are discussed in relation to the main social theories on what drives temperature-related violence.

## Materials and methods

### Study area

**Fig. 1:**
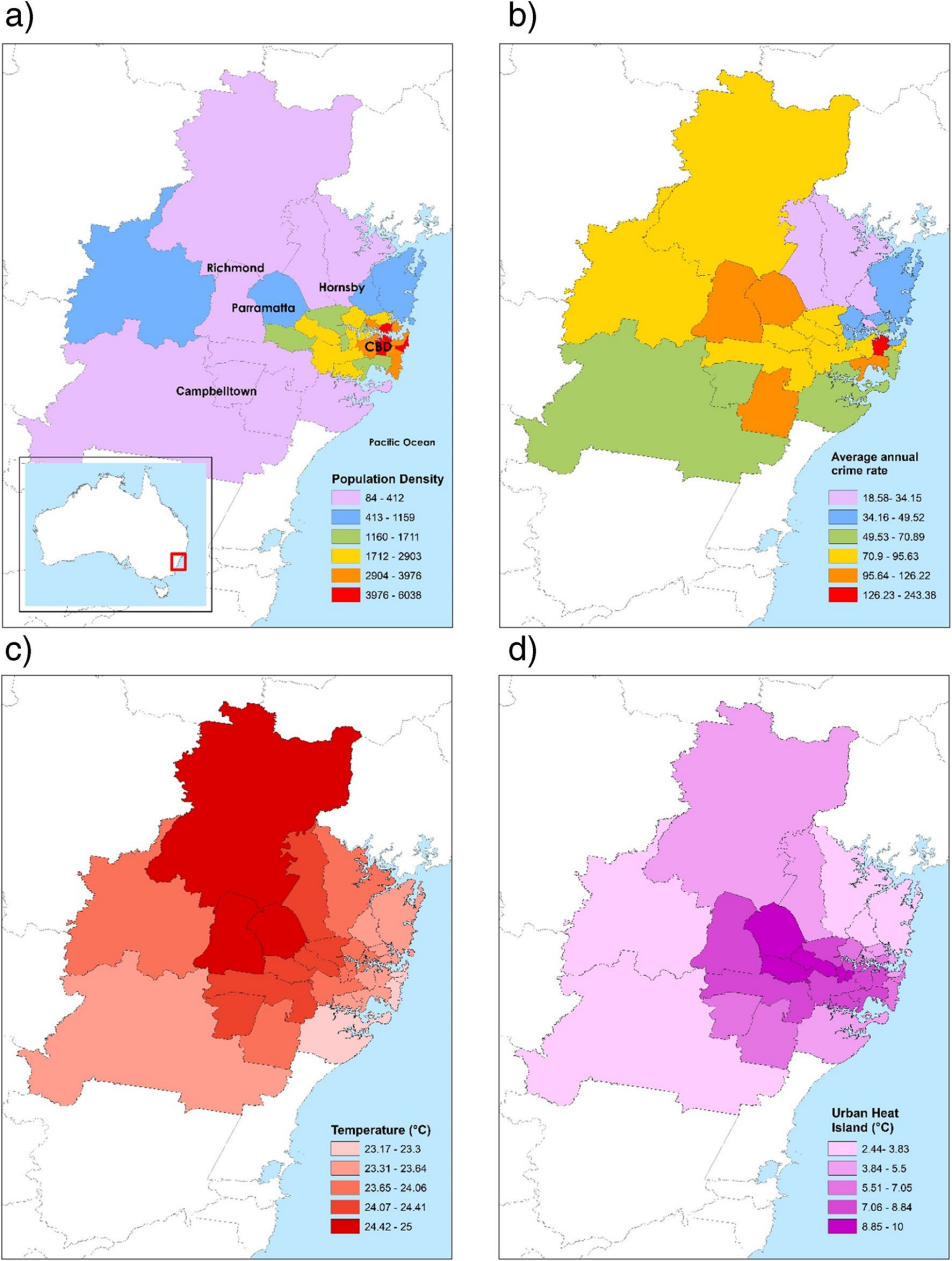

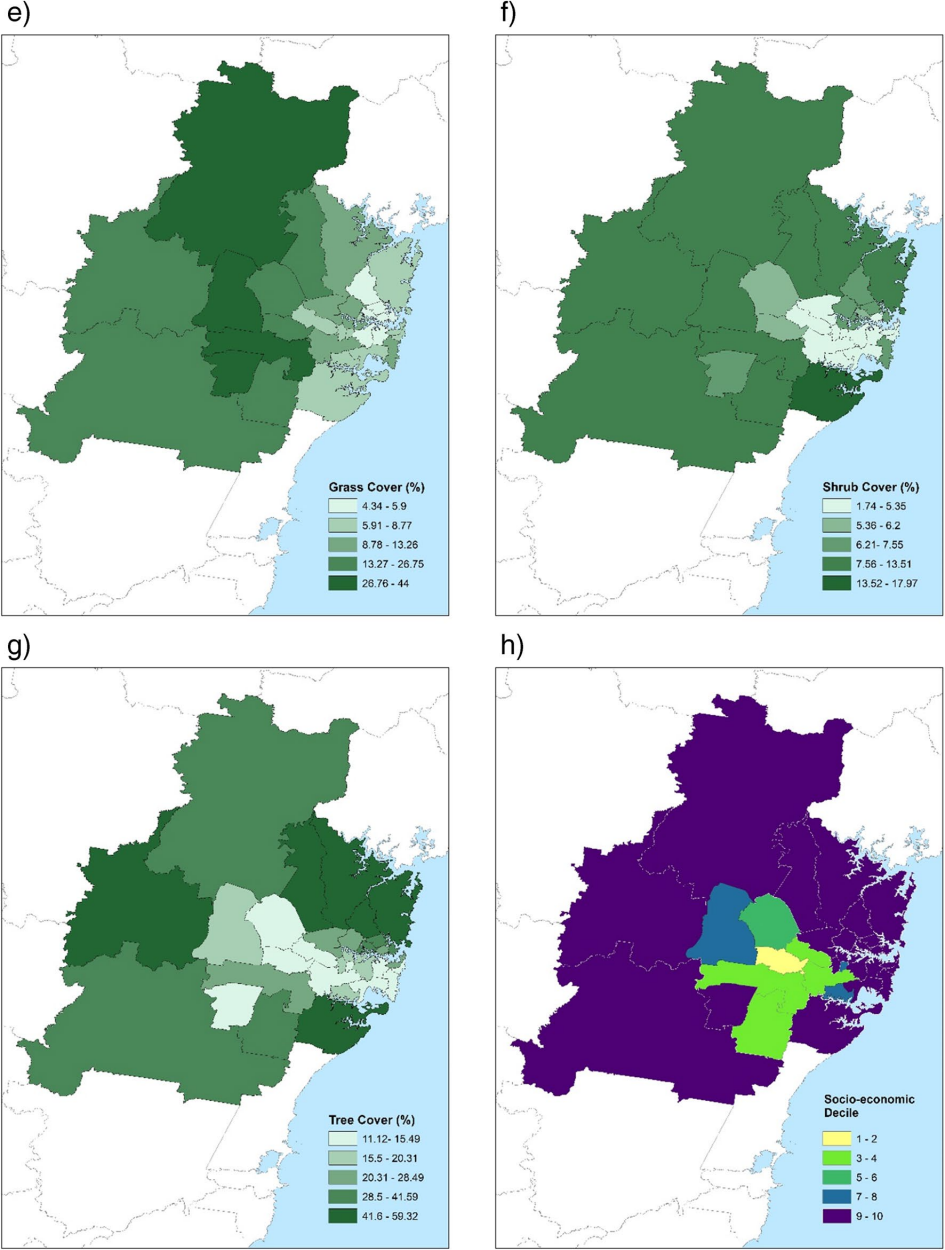
Local government areas of the Greater Sydney Region, showing **a** population density (people per km.^2^), **b** annual average violent crime rate (per 10,000 people), **c** average maximum air temperature (°C), **d** urban heat island magnitude (based on land surface temperature, °C), **e** grass cover (%), **f** shrub cover (%), **g** tree cover (%) and **h** socio-economic index (decile)

This study was of 33 local government areas (LGAs) in the Greater Sydney Region (GSR), the capital city of New South Wales, which is located on the mid-east coast of Australia (Fig. 1a). Local government area refers to a geographical subdivision for which there is a governing council involved in decisions related to greening and housing density. The 2016 LGA boundaries (Australian Bureau of Statistics 2016) were used here to match the data collection period. The GSR sits on a coastal plain of 5063 km^2^ bounded by the Pacific Ocean to the east, the Blue Mountains in the west (1017 m above mean sea level [MAMSL]), and highlands to the south (500–900 MAMSL). The climate is classified as humid subtropical (Köppen climate classification Cfa, Köppen et al. (2011)). The population at the midpoint of the study, in 2016, was 4,823,991, 49.3% male, 50.7% female and median age 36.

### Data

The total number of daily violent crime incidents (defined by the NSW Department of Justice, Bureau of Crime Statistics and Research (BOCSAR) as incidents of domestic assault, non-domestic assault, assault against police, sexual assault and murder) that occurred in each of 33 LGAs within the GSR from 01 July 2013 to 30 June 2018 was obtained from BOCSAR (BOCSAR reference NM1715202). Assault is defined by BOCSAR as the direct (and immediate/confrontational) infliction of force, injury, or violence upon a person or persons or the direct (and immediate/confrontational) threat of force, injury or violence where there is an apprehension that the threat could be enacted (Bureau of Crime Statistics and Research 2020). Assaults were classified as inside if they occurred inside a residence, building, shop or similar (as recorded in the BOCSAR data) and otherwise they were classed as outside. Assaults for which the location was classified as unknown were excluded from analysis.

The average daily maximum temperature was calculated for each LGA using the gridded meteorological datasets from the Australian Water Availability Project (Jones et al. 2009). Surface urban heat island magnitude (°C) was obtained from the CSIRO ‘Land surface temperature and urban heat island estimates for Australian urban centres’ (Caccetta et al. 2017) and available for 2016 only. These values were calculated by subtracting an estimate of non-urban baseline temperature from land surface temperature observations and are a fixed value over the study period. Greencover data were obtained from the GSR urban vegetation cover modified mesh block 2016 (Department of Planning, Industry and Environment, 2019). The data were derived from high-resolution multispectral imagery and provide the percentage of greencover by area blocks (Caccetta et al. 2019). Greencover types are defined by the height of the vegetation in meters, grass < 0.5 m, shrub 0.5 to 3 m and tree > 3 m. These values were also fixed over the study period. Greencover percentage was calculated as the percentage of that type of vegetation in the total land area of a given LGA.

As a measure of socio-economic status (SES), the Index of Relative Socio-economic Disadvantage (IRSD) 2016, from the Socio-Economic Indexes for Areas (SEIFA), was used (Australian Bureau of Statistics 2018). The IRSD is a weighted combination of 16 national census variables relating to the economic and social disadvantages of people and households within an area (such as income, unemployment, home ownership, disability and education). Using the state-based decile, a low score indicated an LGA with relatively greater socio-economic disadvantage. Population for each LGA was also obtained from this dataset in order to estimate crime rate.

### Analysis

For visual presentation, LGA population density (people per km^2^), average annual violent crime rate (per 10,000 people), average maximum ambient air temperature (°C), surface UHI magnitude (based on land surface temperature, °C), grass, shrub and tree cover (%), and socio-economic decile were categorised and displayed on maps of the GSR. Spearman’s correlation was used to measure the strength of the monotonic relationship between variables of interest.

Panelised negative binomial time series generalised additive models (GAMs) were used to estimate the daily violent crime rate with penalised spline terms for the random intercept and slope controlling for repeated measures within each LGA. GAM models were used so that non-linear relationships between each numeric predictor and assault rate could be assessed and incorporated using splines. Predictors for which a non-linear relationship was not indicated (effective degrees of freedom equal to 1) were incorporated using a linear term. Poisson’s family models were also considered, but the crime data displayed overdispersion; therefore, a negative binomial GAM was ultimately utilised. Analysis was undertaken separately according to location classification (inside or outside) for all assaults as well as separately by crime type (domestic violence, non-domestic violence and sexual assault). Incidents of murder were too few to allow separate analysis. The time series models incorporated an effect for trend over time, seasonality (via season), temperature and socio-economic disadvantage (via the IRSD decile) as well as the surface UHI and measures of green space, described earlier. Interactions between season and surface UHI or green space were checked and retained if significant. The purpose of the interaction was to determine whether surface UHI or green space effects were persistent or had differential impact by season. Results are presented as coefficient with standard error (SE) and *p*-value. Significance was taken to be *p*-values < 0.05.

All analyses were completed using R version 4.2.2 statistical software (R Core Team 2022) within RStudio version 2023.03.1. The package ‘mgcv’ (Wood 2011) was used for time series modelling. Spatial maps were generated using ArcGIS Desktop version 10.7.1 (Environmental Systems Research Institute, 2019).

## Results

### Descriptive statistics

In the Greater Sydney Region, LGA area size ranged between 8.6 and 748.8 km^2^ (mean 206.9 km^2^, ± 237.6 km^2^), with larger LGAs typically found in the peripheral areas (Fig. 1a). The overall population density was 650 people per km^2^ and LGAs with the highest density were to the east, around the central business district (CBD) area (Fig. 1a).

A total of 182,962 incidents of violent crime occurred in the study period, of which 48.0% were non-domestic assault (*n* = 87,860), 39.7% domestic assault (*n* = 72,687), 12.1% sexual assault (*n* = 22,121) and 0.2% murder or murder-related (*n* = 294). The majority of assaults occurred inside (71.1%, *n* = 130,016). The violent crime rate was 82.5 incidents per 10,000 people per year (ranging in LGA from 18.6 to 243.4) (Fig. 1b). Higher assault rates were concentrated around central and inner western LGAs.

The mean of the LGA average maximum temperature during the study period was 23.9 °C (± 0.5 °C, range 23.2 to 25.0 °C). Temperatures increased with distance from the coast but slightly decreased as topography rose in the far west and south (Fig. 1c). The mean surface UHI was 6.7 °C (± 2.3, range 2.4 to 10.0). Surface UHI closely aligns with population density, being highest in the CBD and through the central west (Fig. 1d).

The average percentage of LGA area with green space cover in the GSR was 48.6% (range 21.0 to 88.0%). Of all greencover in the GSR, the most common was tree (53.7%, 1862km^2^), with the percentage of LGA area being tree cover ranging from 11.1 to 59.3% (Fig. 1g). LGAs with high tree cover were predominately found in the peripheral northern, western and southern areas where there are large areas of public forest. The next most common was grass cover (31.2%, 1082km^2^), with the percentage of LGA area being grass cover ranging from 4.3 to 44.0% (Fig. 1e). High grass cover was largely found in the west and northwest, in areas that had farms and/or flood plains. Shrubs made up 15.1% of greencover (523km^2^), with the percentage of LGA area being shrub cover ranging from 1.7 to 18.0% (Fig. 1f). LGAs with higher shrub cover percentages were largely found in the peripheral LGAs.

At the LGA level, the decile for Index of Relative Socio-economic Disadvantage was generally high (less disadvantage) with a median of 10 (mean 8, range 1 to 10) (Fig. 1h). As a state-ranked decile, the median is disproportionately high due to the GSR being the state capital city. Lower deciles were generally found in the central LGAs while a higher index was found elsewhere.

#### Correlations

**Fig. 2:**
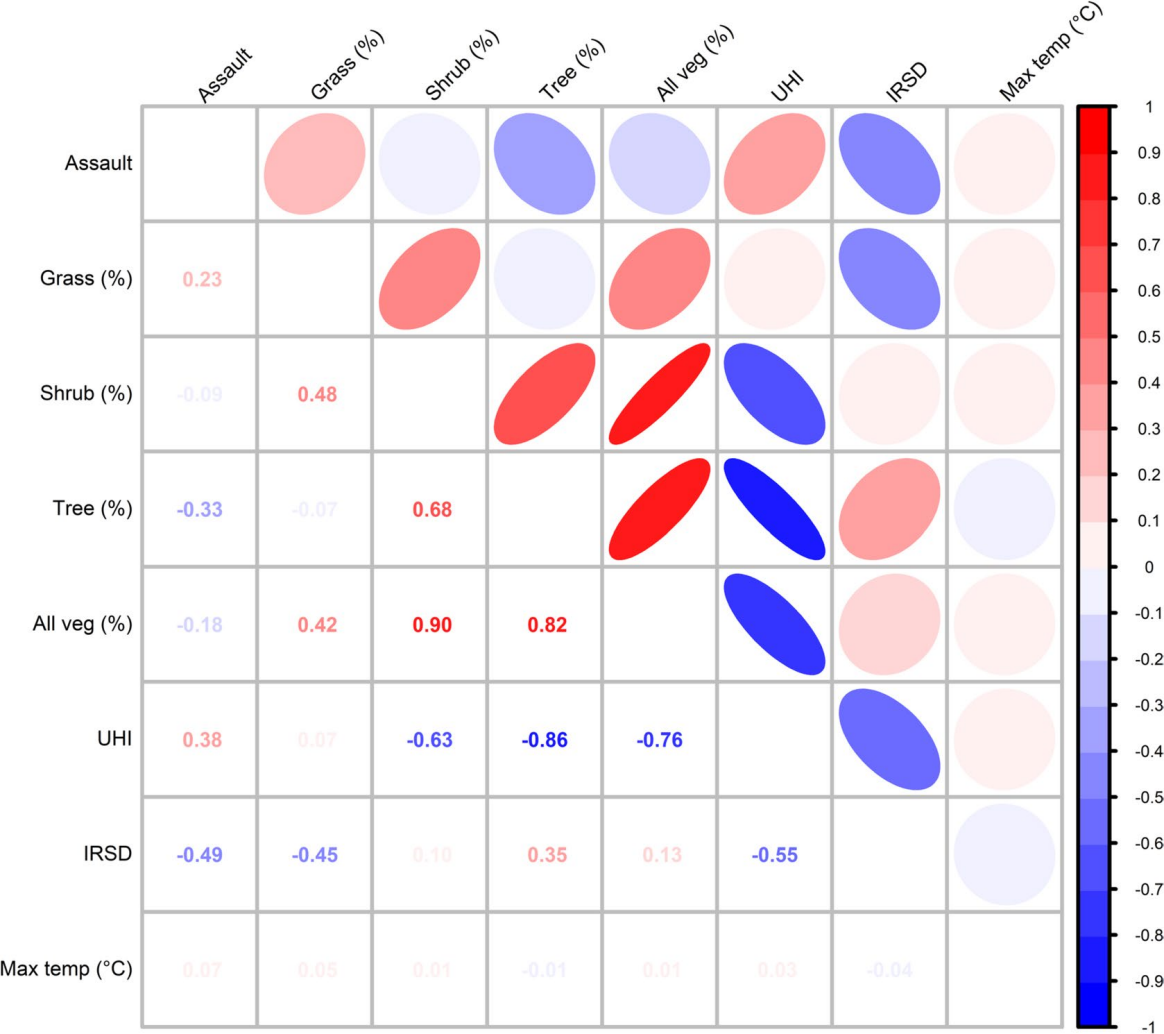
Spearman correlation between local government area daily violent crime frequency and environmental and demographic variables for the Greater Sydney Region

Pairwise Spearman’s correlations between daily frequency of assault, average maximum temperature, IRSD decile and percentage of grass, shrub and tree cover and all vegetation are presented in Fig. 2. Results showed that increasing frequency of violent crimes was moderately correlated with lower percentage of tree cover, higher surface UHI and increasing socio-economic disadvantage (lower IRSD decile) (*r* =  − 0.33, 0.38, − 0.49, respectively, *p* < 0.001). Increasing average surface UHI magnitude was highly correlated with decreasing proportions of shrub, tree and all vegetation cover percentage (*r* <  − 0.6), precluding their use in the same time series model. Surface UHI magnitude was also moderately correlated with higher socio-economic disadvantage (*r* =  − 0.55, *p* < 0.001). Higher socio-economic disadvantage was moderately correlated with increasing percentage of grass cover (*r* =  − 0.45, *p* < 0.001). Because shrub and tree cover percentage were very highly correlated with all vegetation percentage (*r* > 0.8, *p* < 0.001), we used only all vegetation percentage in the time series models to avoid multiple testing issues.

### Panelised time series models

The coefficients for each of the models for all assaults are shown in Table 1. In general, there was no evidence of a change over time in assault rates (*p* > 0.06) for any model except for the inside surface UHI model which suggested weak evidence of a decrease over time (*p* = 0.049). In most models, spring, summer and autumn had significantly more violent crime than winter for crimes that occurred inside (*p* < 0.03). Increasing average maximum temperature was associated with increasing violent crime (*p* < 0.001) for crimes committed both inside and outside. The association between temperature and crime rate was typically non-linear (see Supplementary Fig. 1) with rates increasing to about 30 °C and then decreasing again.

**Table 1:** Panelised negative binomial time series model coefficients with standard error (SE) and *p*-value for predicting local government area (LGA) violent crime rate by location

Violent crime type	All	
Location	Inside	Outside
Urban heat index models		
Intercept	− 2.00 (0.51); *p* < 0.001	− 3.81 (0.57); *p* < 0.001
Time	− 0.05 (0.03); *p* = 0.049	− 0.01 (0.03); *p* = 0.566
Season (ref = winter)		
Spring	0.06 (0.01); *p* < 0.001	0.04 (0.06); *p* = 0.507
Summer	0.11 (0.01); *p* < 0.001	0.13 (0.06); *p* = 0.025
Autumn	0.05 (0.01); *p* < 0.001	− 0.02 (0.06); *p* = 0.756
UHI (°C)	0.07 (0.04); *p* = 0.072	0.13 (0.05); *p* = 0.006
IRSD decile	− 0.07 (0.04); *p* = 0.038	− 0.00 (0.04); *p* = 0.904
Maximum temperature (per 10 °C)	0.09 (0.01); *p* < 0.001	See supp Fig. 1A; *p* < 0.001
UHI (°C)$\times$Season	NA; *p* = 0.404*	*p* = 0.006
UHI$\times$Spring		− 0.00 (0.01); *p* = 0.694
UHI$\times$Summer		− 0.02 (0.01); *p* = 0.002
UHI$\times$Autumn		− 0.00 (0.01); *p* = 0.838
Grass cover models		
Intercept	− 1.51 (0.32); *p* < 0.001	− 2.41 (0.40); *p* < 0.001
Time	− 0.05 (0.03); *p* = 0.060	− 0.01 (0.03); *p* = 0.581
Season (ref = winter)		
Spring	0.04 (0.02); *p* = 0.032	0.02 (0.03); *p* = 0.556
Summer	0.07 (0.02); *p* < 0.001	0.02 (0.03); *p* = 0.528
Autumn	0.02 (0.02); *p* = 0.307	− 0.02 (0.03); *p* = 0.513
Grass cover (per 10%)	0.13 (0.08); *p* = 0.097	− 0.03 (0.10); *p* = 0.745
IRSD decile	− 0.09 (0.03); *p* = 0.002	− 0.07 (0.04); *p* = 0.091
Maximum temperature (°C)	See supp Fig. 1B; *p* < 0.001	See supp Fig. 1C; *p* < 0.001
Grass (per 10%)$\times$Season	*p* = 0.039	*p* = 0.035
Grass$\times$Spring	0.01 (0.01); *p* = 0.145	0.00 (0.01); *p* = 0.973
Grass$\times$Summer	0.03 (0.01); *p* = 0.007	− 0.04 (0.02); *p* = 0.013
Grass$\times$Autumn	0.02 (0.01); *p* = 0.024	− 0.01 (0.02); *p* = 0.546
All vegetation models		
Intercept	− 1.08 (0.32); *p* < 0.001	− 2.04 (0.35); *p* < 0.001
Time	− 0.05 (0.03); *p* = 0.062	− 0.01 (0.03); *p* = 0.572
Season (ref = winter)		
Spring	0.06 (0.01); *p* < 0.001	0.02 (0.02); *p* = 0.360
Summer	0.11 (0.01); *p* < 0.001	− 0.03 (0.02); *p* = 0.120
Autumn	0.05 (0.01); *p* < 0.001	− 0.11 (0.02); *p* = 0.093
All vegetation (per 10%)	− 0.03 (0.04); *p* = 0.430	− 0.11 (0.04); *p* = 0.011
IRSD decile	− 0.11 (0.03); *p* < 0.001	− 0.05 (0.03); *p* = 0.145
Maximum temperature (°C)	See supp Fig. 1D; *p* < 0.001	See supp Fig. 1E; *p* < 0.001
All vegetation (per 10%) ‘ Season	NA; *p* = 0.334*	NA; *p* = 0.699*

*UHI*, urban heat index (based on land surface temperature, °C); *IRSD*, index of relative socio-economic disadvantage (higher values = less disadvantage); all models include a random intercept and slope for LGA and an offset for LGA population; *non-significant interactions were removed from the model

Significant *p* values shown in bold

Surface UHI was positively associated with violent crimes that occurred outside (*p* = 0.006), with higher surface UHI having more impact on assault rates in winter versus summer *(p* = 0.002). Similar patterns were seen when violent crimes were separated by crime type (see Supplementary Table 1, *p* < 0.04).

More grass cover was associated with increased inside violent crime rates in summer (*p* = 0.007) and autumn (*p* = 0.024) compared to winter and lower outside rates in summer (*p* = 0.013). When analysed by crime type, more grass cover was associated with increased inside domestic and sexual assaults only (*p* ≤ 0.012, Supplementary Table 1).

Increased all vegetation cover was associated with decreasing violent crime rates for crimes that occurred outside (*p* = 0.011) but was not associated with violent crimes committed inside (*p* = 0.430). Similar patterns were observed for domestic and non-domestic violent crimes (Supplementary Table 1).

Increased socio-economic disadvantage (lower deciles) was associated with increased violent crime for crime that occurred inside for all models (*p* < 0.04) but not outside (*p* > 0.09). When separated by crime type, a similar pattern was observed for domestic assaults committed inside (*p* ≤ 0.013), sexual assaults committed inside for the all vegetation model (*p* = 0.019) and for the greencover models with domestic assaults committed outside (*p* < 0.02).

## Discussion

This study brings together several fields of research often considered separately: crimes by temperature, crime by green space, temperature by green space and crimes by type and premises. Combining these fields provides a better understanding of the associations between violent crime and urban temperature modulation from UHI or greencover. This study is the first to find a significant association between violent crimes that occurred outside and the urban heat island effect and expands our understanding of crime by greencover.

### Acute and chronic temperature effects on violence

This study found that outside violent crime significantly increased with the surface UHI and decreased with more vegetation. It also found that increasing average maximum temperature was associated with increasing violent crime (*p* < 0.001) for crimes committed both inside and outside and was higher in summer compared to winter. Together, this could suggest that there might be both *acute* and *chronic* temperature effects on violent crime. For example, a summer or hot day represents an acute exposure that occurs then recedes, and all areas (generally) experienced the effect. The UHI and greencover however represent a ‘chronic’, or permanent heat modifier, in that areas remain hotter or cooler relative to other areas (noting that there is variance over time and space in the magnitude of both the UHI (Santamouris 2015) and greencover (Ossola et al. 2021)).

### Effects vary by crime location and type

This study found that violent crime was associated with surface UHI and vegetation generally only for assaults that occurred outside (not in). Surface UHI is associated with increasing violent crime, while vegetation is associated with less. This generally remains true by crime type—all crime types increased with surface UHI if they occurred outside; however, non-domestic violence also increased inside with surface UHI. By vegetation, there was less domestic and non-domestic violence outside in LGAs with increasing vegetation cover; however, there was no association with sexual assault, regardless of location. These associations can be explored through considering the theory on temperature-related violence.

### Theories on temperature-related violence

The General Aggression Model (GAM) is a useful overarching framework that proposes an act of aggression includes three phases; *inputs* (being person or situational) influence a person’s *internal state* which in turn influences *outcomes*. For example, in warm temperatures (the input), people increase their socialisation and alcohol consumption (affecting internal state), potentially increasing motivations and opportunities for aggression (outcomes). (Felson 2016). However, in uncomfortable temperatures, the drivers change; for example, consider the Western Sydney LGA of Penrith, which had the highest average maximum temperature, high surface UHI effect (8.29 °C) and low tree cover (19%), and in 2019/2020 experienced the historical record high air temperature of 48.9 °C (Tabassum et al. 2021). Radiant heat from the carparks was measured at 80 °C and the surface temperature from playground equipment peaked at 100 °C (Purtill 2021). Applying the GAM framework, temperature would affect people physically (i.e. sweating, burns, lethargy) as well as altering behaviour such as retreating indoors, both of which could cause a negative internal state and result in increased likelihood of violence. In these conditions, it is possible people may ‘escape’ to cooler environments like retreating indoors or visiting shopping centres, as proposed by the negative affect escape model (Bell 1992). This may explain why this study found crimes that occurred inside were generally not associated with surface UHI or all vegetation.

Furthermore, routine activity (RA) theory proposes that crime occurs when circumstances bring motivated offenders and possible victims into contact, in the absence of guardians (Cohen & Felson 1979). During ‘warm’ summer temperatures, it is possible that people congregate in cool areas (e.g. leafy parks, coastal areas) and this increased social interaction provides opportunities for violence. This study visualised crime rates, seeing higher rates in inner-city coastal LGAs (i.e. Bondi, Coogee). It is possible that people visit cooler areas for recreation or tourism and elevate crime rates. Similarly, higher rates were found in LGAs that host sports or races (i.e. City of Sydney, Parramatta); these areas also often have areas of grass cover. In extreme heat however, routines change as people move indoors to avoid the heat; RA theory would suggest that this also creates opportunities for motivated offenders and lack of guardians. Being indoors shields people from the natural surveillance of ‘eyes on the street’ (as coined by Jacobs (1961)), and creates opportunities to act violently towards partners or children. The absence of witnesses to an aggressive act reduces the likelihood of the crime being reported, and subsequent retribution for the offender. Rotton and Cohn (2004) considered violent crime by if the premises where it occurred was likely air-conditioned, finding that while the likelihood of assaults increased with temperature; in high temperatures, assaults outside declined while assaults inside continued to increase. The results of this study support the findings of Rotton and Cohn. This variation in behaviour by temperature range may explain the non-linear trend that has been demonstrated in numerous other temperature-crime studies (Bell & Fusco 1989; Rotton & Cohn 2000; Xu et al. 2020).

### Greencover; cooling temperatures and tempers

It may be that cooling the person mitigates the effect of temperature on aggressive behaviour; controlled studies have found that having a cold drink or accessing air-conditioning reduces the impacts of temperature on aggressive behaviour (Baron & Bell 1976; Kenrick & MacFarlane 1986). Green space reduces both ambient air temperatures as well as land surface temperatures (Bowler et al. 2010) and therefore may reduce temperature-related violence by creating cooler environments. Indeed, this study found that the proportion of shrub and tree cover had a moderate to high negative correlation with surface UHI. As well as climate modulation, there are many other causal mechanisms that could determine causality between green space and reduced crime including social interaction and recreation, opportunities and motivations for physical activity, community perception, biophilic stress reduction, and a perceived sense of order (‘cues to care’) (Donovan & Prestemon 2010; Hartig et al. 2014; Shepley et al. 2019; Troy et al. 2012). Overall, a multi-disciplinary research synthesis concluded ‘The balance of evidence indicates conclusively that knowing and experiencing nature makes us generally happier, healthier people’ (page 473, Russell et al. (2013)).

Grass cover, however, had somewhat different trends to all vegetation; there was a moderate negative correlation with SES (while tree cover had a positive association) and negligible correlation with surface UHI. It may be that activities that occur in areas with high grass cover are mixed. For example, the LGAs with high amount of grass tended to have large sporting grounds, parks or racetracks, which may host sporting or social events that can temporarily inflate the population of that area and also be associated with increased violence. Parks have also been called ‘social holes’ with a lack of guardianship leading to more crime (Hipp et al. 2014). However, in the GSR, LGAs with higher grass cover were also farmland in the western areas of the study area which may attract different types of behaviour.

### The disproportionate effects on the disadvantaged

This study highlights inequalities by SES: LGA SES had a moderate negative correlation with assault rates. Lower SES LGAs tended to have decreasing tree cover and increasing surface UHI. This may, in part, explain why studies have found the effect of temperature on violence is more pronounced in low SES populations (Harries et al. 1984; Otrachshenko et al. 2021). LGAs with more disadvantage also typically had higher population density. This is significant given Griffit and Veitch (1971) found higher population density alongside increasing temperature reduced interpersonal affective behaviour (people liking each other).

### Can greencover mitigate current and future temperature-related violence?

While increasing green space may reduce temperature-related violence, the authors agree with Bogar and Beyer (2016), who note ‘it is unlikely that green space alone will suffice as an intervention to community violence and crime, both of which have vastly complex roots’ (pg. 169). Rather, green space and the vegetated areas therein should be considered holistically, and in combination with other urban cooling measures like water design features, lighter surface colours and air flow (Mohajerani et al. 2017; Oke et al. 2017). On temperature-related crime reduction, green space is just one measure that sits within a broader suite of interventions at the scale of the individual (e.g. managing alcohol consumption, retrofitting houses for cooling), relationships (e.g. couples and parenting education) and societies (e.g. public awareness programs and targeted police/health/urban planning strategies).

While this study found more vegetation was associated with less crime, green space may also reduce future violence relating to climate change. The average global temperature is now more than 1 °C above pre-industrial levels and the years 2015–2019 were the five warmest on record (World Meteorological Organization 2021). Australia is projected to experience more frequent, longer and hotter extreme heat events (Argüeso et al. 2015). A growing number of studies suggest climate change will increase violent crime. Burke et al. (2015) found a 1 standard deviation change in climate variables results in a 4% change in interpersonal violence, while Ranson (2014) projected that between 2010 and 2099, climate change will cause an additional 22,000 murders, 180,000 incidents of rape and 3.5 million additional assaults in the USA alone. Urban planning and climate change adaptation efforts are integrating nature-based, social and technical tools to address city resilience (Lin et al. 2021) and it may be that crime mitigation efforts could also make use of a similarly integrated approach.

### Limitations

This study uses Australian data, and as such some findings may not fully translate to Northern Hemisphere environments. Crime trends in Australia are different; for example, in 2017, the share of deaths from interpersonal violence was 0.19% (1.4 homicides per 100,000 people), while in the USA it was 0.70% (6.1 per 100,000 people) and in the UK 0.06% (0.5 per 100,000 people) (Institute for Health Metrics Evaluation, 2017). Regarding greencover, the native vegetation in Australia is generally not deciduous, so vegetation appearance varies little with seasons.

## Conclusion

This study explored the nexus between several critical issues; interpersonal violence (including domestic and family violence), urban densification and form, and increasing temperatures. Overall, the study found that increasing average maximum temperature was associated with increasing violent crime for crimes committed both inside and outside. However, only crimes that occurred outside were associated with increasing surface UHI and decreasing vegetation cover. While the drivers of temperature and violent crime are complex, green space has the ability to make areas cooler and more comfortable and usable in heat events. This study is the first to consider the effect of urban form, both the urban heat island effect and urban greencover, on temperature-related violence, and the findings are particularly valuable considering rapid changes in urban population, urban density, and climate change.

### Supplementary Information

Below is the link to the electronic supplementary material.

Supplementary file1 (DOCX 129 KB)

Supplementary file2 (DOCX 269 KB)

Supplementary file3 (DOCX 21 KB)
