# Supplementary material for: Associations between violent crime inside and outside, air temperature, urban heat island magnitude and urban green space
Source: Int J Biometeorol. 2024 Jan 8;68(4):661–73. doi: 10.1007/s00484-023-02613-1 (PMC10963557; doi:10.1007/s00484-023-02613-1)

**SUPPLEMENTARY FIGURE 1** Smooth curves (splines) for average maximum temperature for the models in Table 1.

A) UHI and outdoor assault model. Relationship between assault and temperature.


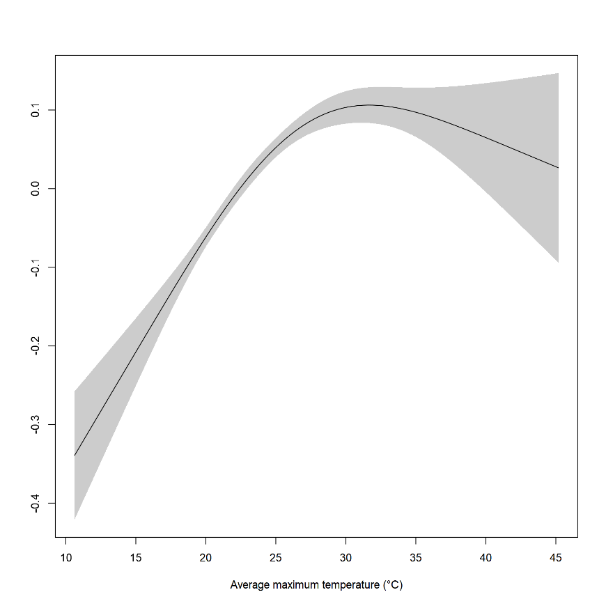


B) Grass cover and indoor assault model. Relationship between assault and temperature.


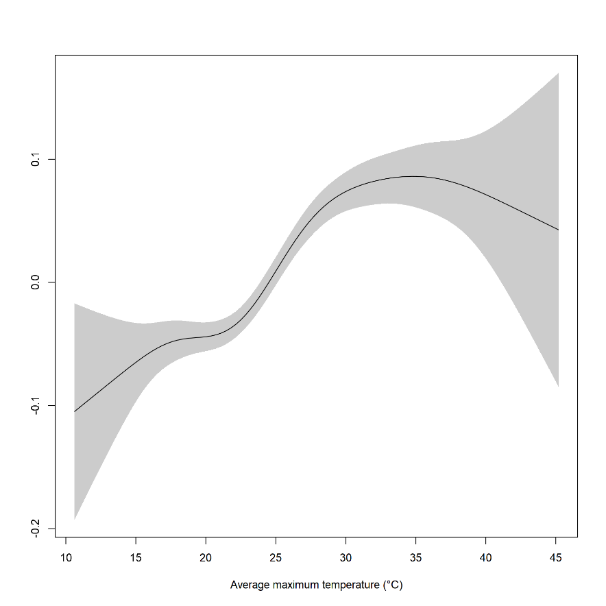


C) Grass cover and outdoor assault model. Relationship between assault and temperature.


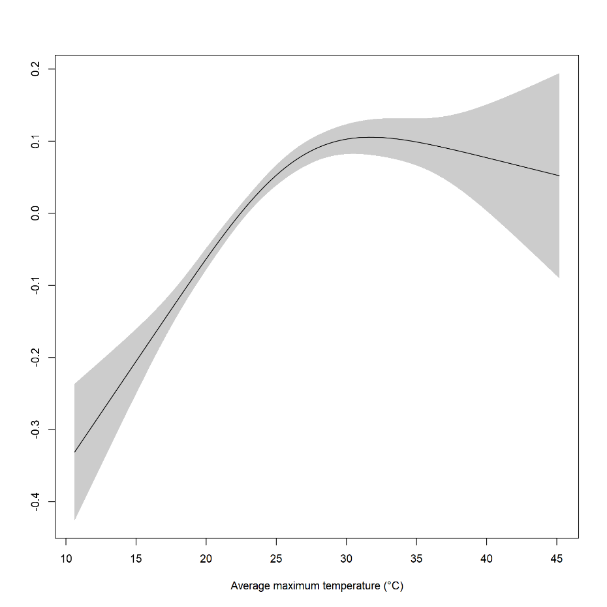


D) All vegetation cover and indoor assault model. Relationship between assault and temperature.


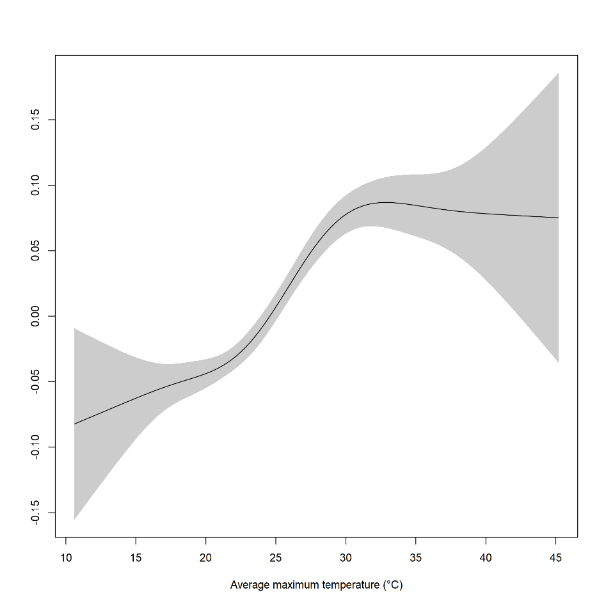


E) All vegetation cover and outdoor assault model. Relationship between assault and temperature.


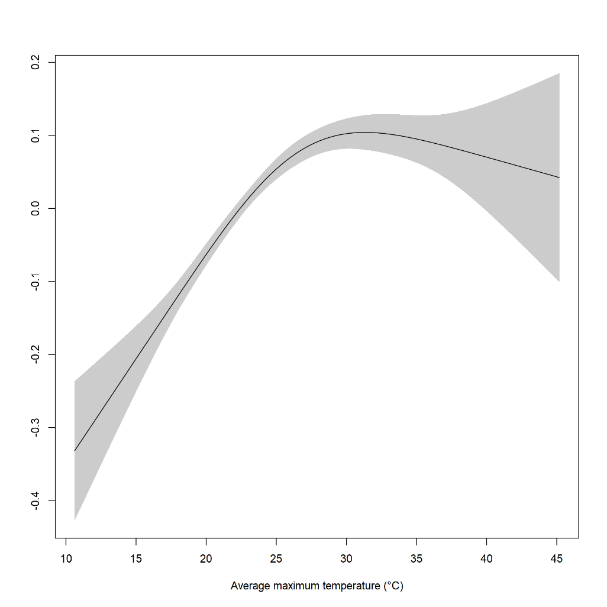

Supplement: Supplementary file 1 — Supplementary file1 (DOCX 129 KB) [file 484_2023_2613_MOESM1_ESM.docx]
