# Supplementary material for: Associations between violent crime inside and outside, air temperature, urban heat island magnitude and urban green space
Source: Int J Biometeorol. 2024 Jan 8;68(4):661–73. doi: 10.1007/s00484-023-02613-1 (PMC10963557; doi:10.1007/s00484-023-02613-1)

**SUPPLEMENTAY FIGURE 2** Smooth curves (splines) for predictor by crime type for the models in Supplementary Table 1.

UHI and indoor [A] (left) or outdoor [B] (right) non-domestic assault models. Relationship between assault and temperature.

| 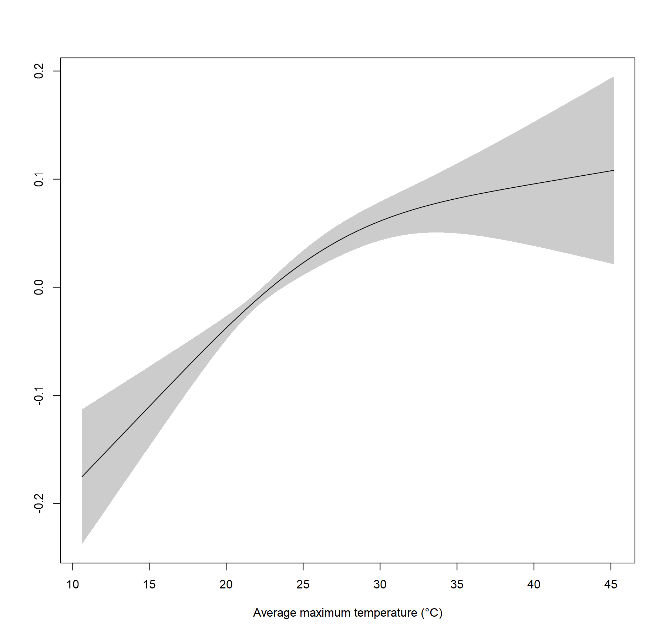 | 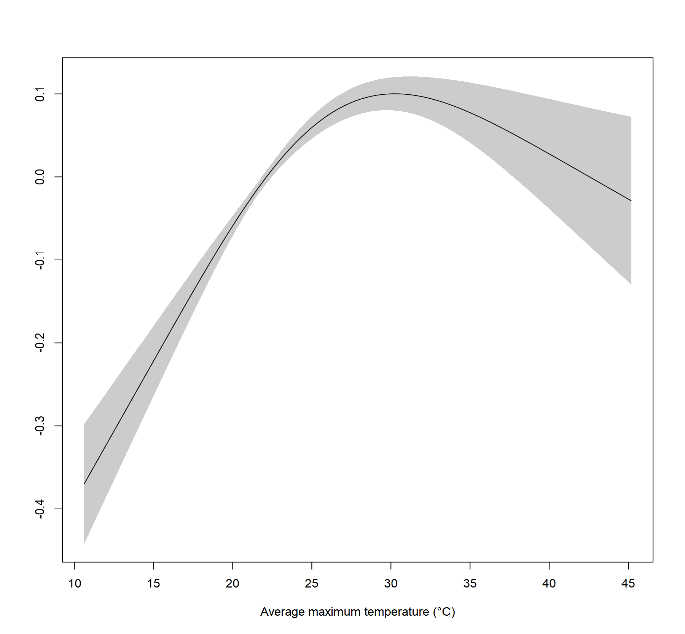 |
| --- | --- |

C) UHI and outdoor sexual assault model. Relationship between assault and temperature.


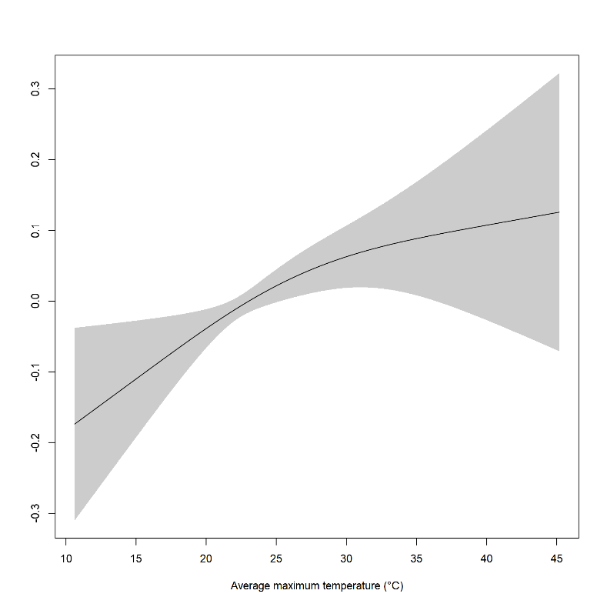


D) Grass cover and outdoor domestic assault model. Relationship between assault and temperature.


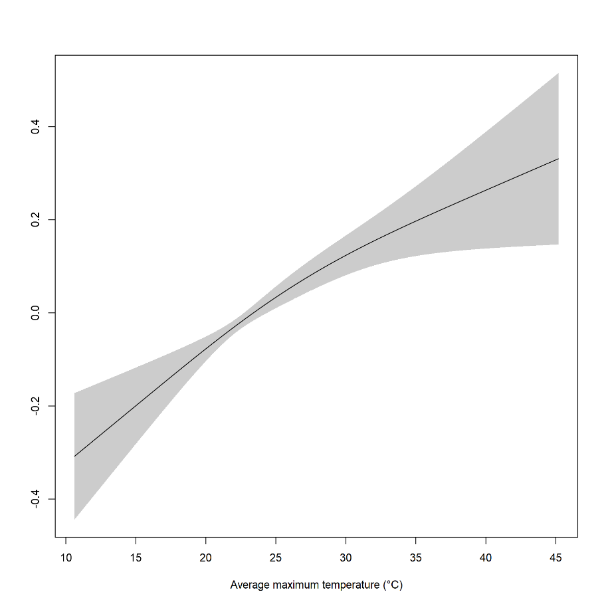


Grass cover and indoor [E] (left) or outdoor [F] (right) non-domestic assault models. Relationship between assault and temperature.

| 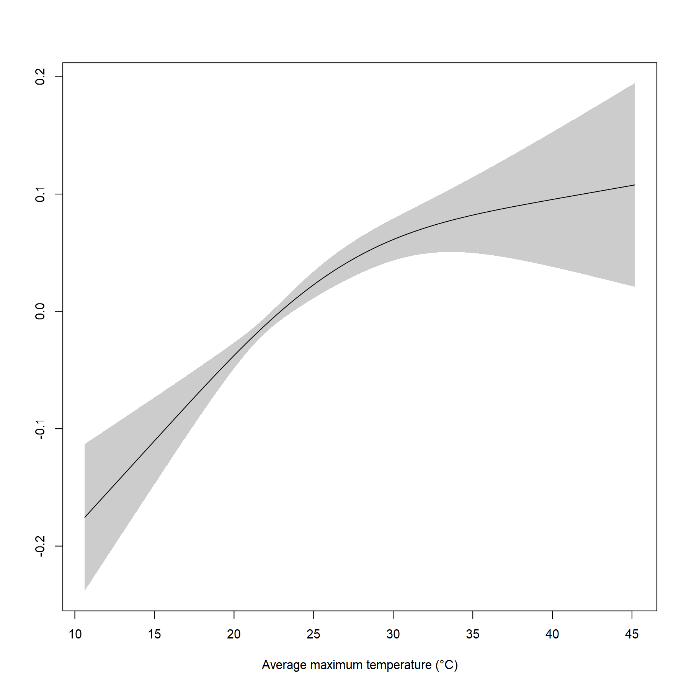 | 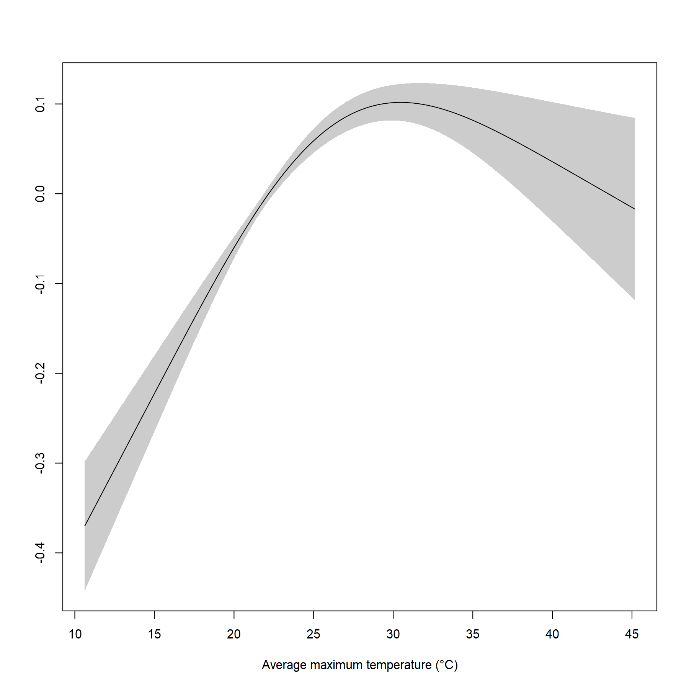 |
| --- | --- |

G) Grass cover and outdoor sexual assault model. Relationship between assault and temperature.


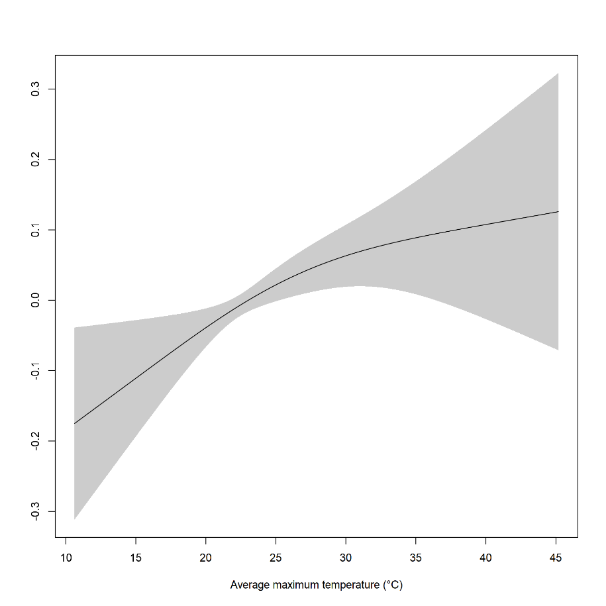


All vegetation cover and indoor [H] (left) or outdoor [I] (right) domestic assault models. Relationship between assault and IRSD decile (left), assault and temperature (right).

| 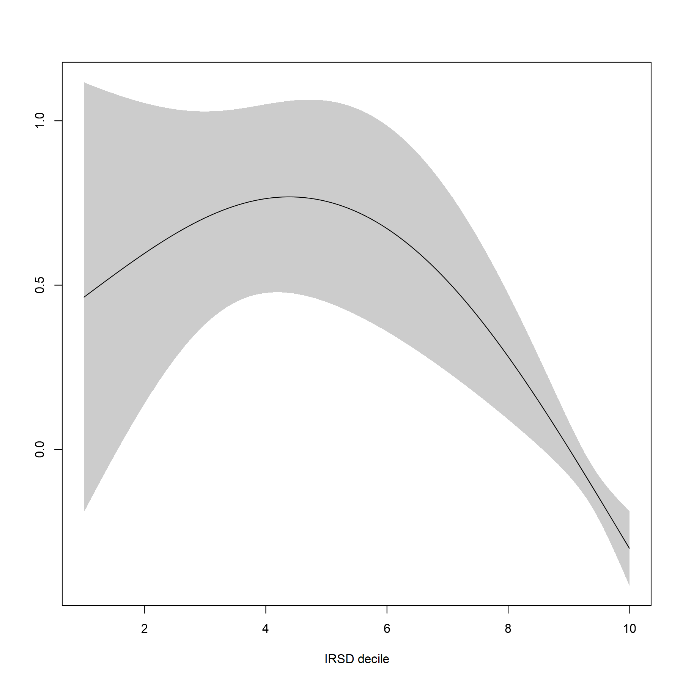 | 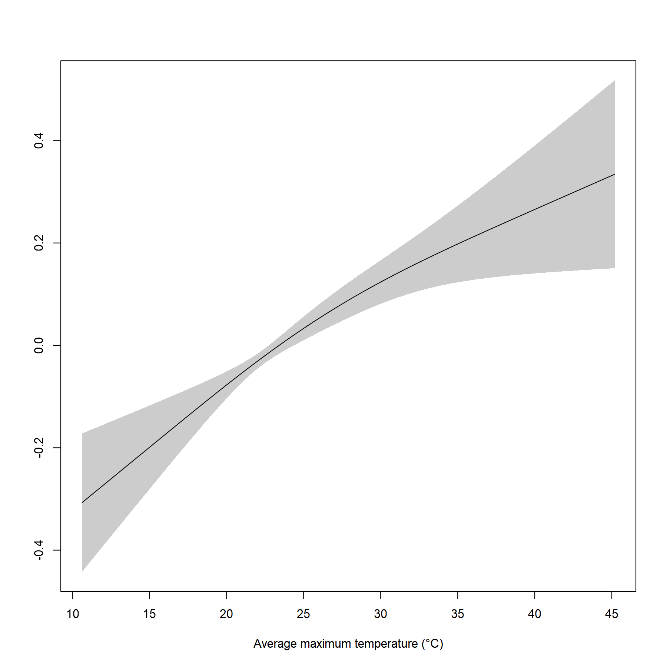 |
| --- | --- |

All vegetation cover and indoor [J] (left) or outdoor [K] (right) non-domestic assault models. Relationship between assault and temperature.

| 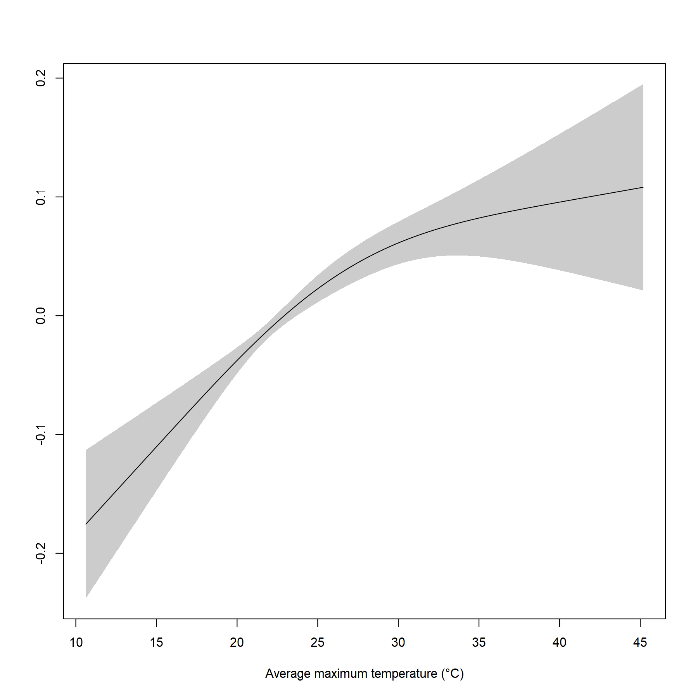 | 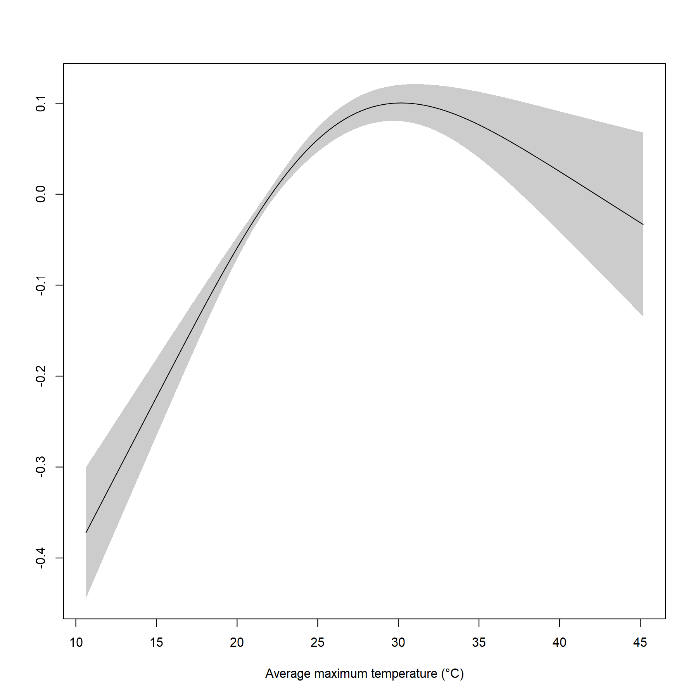 |
| --- | --- |

L) All vegetation cover and outdoor sexual assault model. Relationship between assault and temperature.


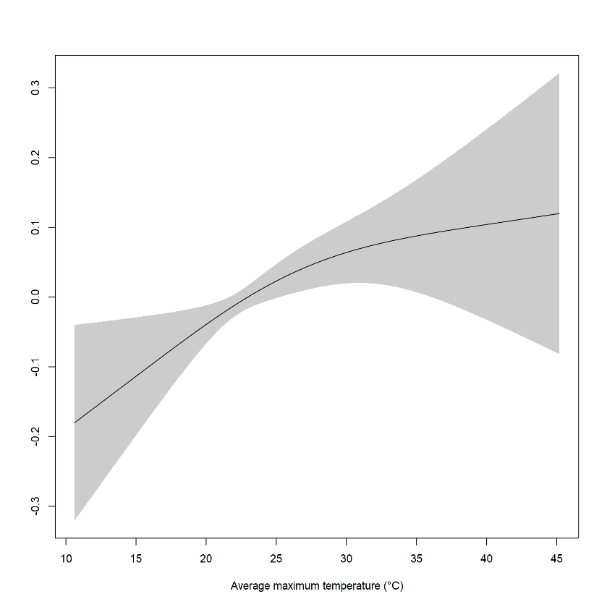

Supplement: Supplementary file 2 — Supplementary file2 (DOCX 269 KB) [file 484_2023_2613_MOESM2_ESM.docx]
