# Supplementary material for: Associations between violent crime inside and outside, air temperature, urban heat island magnitude and urban green space
Source: Int J Biometeorol. 2024 Jan 8;68(4):661–73. doi: 10.1007/s00484-023-02613-1 (PMC10963557; doi:10.1007/s00484-023-02613-1)
Supplement: Supplementary file 3 — Supplementary file3 (DOCX 21 KB) [file 484_2023_2613_MOESM3_ESM.docx]

**SUPPLEMENTARY TABLE 1** Negative binomial time series panel model coefficients with standard error (SE) and p-value for predicting violent crime rate type by violent crime type and location

| **Violent crime type** | **Domestic** | | **Non-domestic** | | **Sexual Assault** | |
| --- | --- | --- | --- | --- | --- | --- |
| **Location** | **Inside** | **Outside** | **Inside** | **Outside** | **Inside** | **Outside** |
| **Urban heat index models** |  |  |  |  |  |  |
| Intercept | **-2.68 (0.52); p<0.001** | **-6.26 (0.60); p<0.001** | **-3.58 (0.60); p<0.001** | **-4.17 (0.61); p<0.001** | **-3.82 (0.48); p<0.001** | **-5.63 (0.47); p<0.001** |
| Time | -0.06 (0.04); p=0.085 | -0.01 (0.05); p=0.779 | -0.01 (0.03); p=0.832 | -0.03 (0.02); p=0.275 | **-0.11 (0.05); p=0.021** | 0.02 (0.07); p=0.744 |
| Season (ref=winter)    Spring    Summer    Autumn | **0.04 (0.01); p=0.013**  **0.11 (0.02); p<0.001**  **0.04 (0.01); p=0.006** | **0.12 (0.04); p=0.005**  0.09 (0.05); p=0.077  0.04 (0.04); p=0.367 | **0.05 (0.03); p=0.010**  **0.05 (0.02); p=0.016**  0.01 (0.02); p=0.497 | -0.01 (0.07); p=0.928  0.12 (0.07); p=0.084  -0.02 (0.07); p=0.806 | **0.13 (0.03); p<0.001**  **0.21 (0.03); p<0.001**  **0.12 (0.03); p<0.001** | 0.01 (0.05); p=0.758  -0.03 (0.05); p=0.518  -0.08 (0.05); p=0.089 |
| UHI (°C) | 0.08 (0.04); p=0.066 | **0.15 (0.05); p=0.002** | **0.10 (0.05); p=0.032** | **0.13 (0.05); p=0.008** | 0.00 (0.04); p=0.943 | **0.08 (0.04); p=0.037** |
| IRSD decile | **-0.11 (0.04); p=0.003** | -0.03 (0.04); p=0.405 | -0.03 (0.04); p=0.474 | -0.00 (0.04); p=0.955 | -0.06 (0.03); p=0.073 | 0.01 (0.03); p=0.659 |
| Maximum temperature (per 10°C) | **0.10 (0.01); p<0.001** | 0.19 (0.03); p<0.001 | **See supp fig 2A; p<0.001** | **See supp fig 2B; p<0.001** | **0.06 (0.02); p=0.014** | **See supp fig 2C; p=0.014** |
| UHI × season    UHI × Spring    UHI × Summer    UHI × Autumn | NA; p=0.613* | NA; p=0.345* | NA; p=0.345* | **p=0.016**  0.00 (0.01); p=0.851  **-0.02 (0.01); p=0.012**  -0.00 (0.01); p=0.890 | NA; p=0.545* | NA; p=0.057 |
| **Grass cover models** |  |  |  |  |  |  |
| Intercept | **-2.29 (0.31); p<0.001** | **-4.36 (0.42); p<0.001** | **-2.62 (0.40); p<0.001** | -2.70 (0.41); p<0.001 | **-4.28 (0.25); p<0.001** | -4.63 (0.31); p<0.001 |
| Time | -0.06 (0.04); p=0.081 | -0.01 (0.05); p=0.835 | -0.01 (0.03); p=0.826 | -0.03 (0.02); p=0.105 | **-0.11 (0.05); p=0.020** | 0.02 (0.07); p=0.754 |
| Season (ref=winter)    Spring    Summer    Autumn | **0.04 (0.01); p=0.012**  **0.11 (0.02); p<0.001**  **0.04 (0.01); p=0.005** | **0.10 (0.05); p=0.022**  0.07 (0.05); p=0.171  0.02 (0.05); p=0.666 | **0.05 (0.02); p=0.010**  **0.05 (0.02); p=0.016**  0.01 (0.02); p=0.499 | 0.02 (0.03); p=0.449  0.03 (0.03); p=0.335  -0.01 (0.03); p=0.652 | **0.13 (0.03); p<0.001**  **0.21 (0.03); p<0.001**  **0.14 (0.03); p<0.001** | 0.01 (0.05); p=0.766  -0.03 (0.05); p=0.510  -0.08 (0.05); p=0.088 |
| Grass cover (per 10%) | **0.19 (0.08); p=0.012** | 0.05 (0.11); p=0.616 | 0.06 (0.10); p=0.518 | -0.03 (0.10); p=0.768 | **0.22 (0.06); p<0.001** | -0.07 (0.08); p=0.365 |
| IRSD decile | **-0.12 (0.03); p<0.001** | **-0.10 (0.04); p=0.019** | -0.07 (0.04); p=0.066 | -0.07 (0.04); p=0.093 | -0.04 (0.02); p=0.120 | -0.03 (0.03); p=0.318 |
| Maximum temperature (per 10°C) | **0.10 (0.01); p<0.001** | **See supp fig 2D; p<0.001** | **See supp fig 2E; p<0.001** | **See supp fig 2F; p<0.001** | **0.06 (0.02); p=0.015** | **See supp fig 2G; p=0.013** |
| Grass cover (%) × season    Grass × Spring    Grass × Summer    Grass × Autumn | NA; p=0.428 | NA; p=0.224 | NA; p=0.077 | **p=0.005**  -0.01 (0.02); p=0.428  **-0.06 (0.02); p=0.001**  -0.01(0.02); p=0.594 | NA; p=0.738 | NA; p=0.386 |
| **All vegetation models** |  |  |  |  |  |  |
| Intercept | **-2.94 (0.20); p<0.001** | **-3.78 (0.38); p<0.001** | **-2.19 (0.37); p<0.001** | **-2.33 (0.37); p<0.001** | **-3.96 (0.28); p<0.001** | **-4.46 (0.028); p<0.001** |
| Time | -0.06 (0.04); p=0.084 | -0.01 (0.05); p=0.830 | -0.01 (0.03); p=0.827 | -0.02 (0.03); p=0.290 | **-0.11 (0.05); p=0.022** | 0.02 (0.07); p=0.755 |
| Season (ref=winter)    Spring    Summer    Autumn | **0.04 (0.01); p=0.012**  **0.11 (0.02); p<0.001**  **0.04 (0.00); p=0.005** | **0.10 (0.05); p=0.022**  0.07 (0.05); p=0.171  0.02 (0.05); p=0.662 | **0.05 (0.02); p=0.010**  **0.05 (0.02); p=0.016**  0.01 (0.02); p=0.499 | -0.01 (0.02); p=0.254  -0.04 (0.02); p=0.071  -0.03 (0.02); p=0.202 | **0.13 (0.03); p<0.001**  **0.21 (0.03); p<0.001**  **0.14 (0.03); p<0.001** | 0.01 (0.05); p=0.790  -0.04 (0.05); p=0.494  -0.08 (0.05); p=0.083 |
| All vegetation (per 10%) | -0.03 (0.04); p=0.490 | **-0.11 (0.05); p=0.017** | -0.07 (0.05); p=0.126 | **-0.12 (0.05); p=0.012** | 0.04 (0.04); p=0.251 | -0.01 (0.03); p=0.606 |
| IRSD decile | **See supp fig 2H; p=0.013** | **-0.09 (0.04); p=0.014** | -0.07 (0.04); p=0.054 | -0.05 (0.04); p=0.175 | **-0.07 (0.03); p=0.019** | -0.08 (0.04); p=0.019 |
| Maximum temperature (°C) | **0.10 (0.01); p<0.001** | **See supp fig 2I; p=0.013** | **See supp fig 2J; p=0.013** | **See supp fig 2K; p=0.013** | **0.06 (0.02); p=0.014** | **See supp fig 2L; p=0.013** |
| All vegetation cover (%) × season | NA; p=0.571 | NA; p=0.888 | NA; p=0.345 | NA; p=0.693 | NA; p=0.970 | NA; p=0.219 |

UHI = urban heat index (based on land surface temperature, °C); IRSD = index of relative social disadvantage (higher values = less disadvantage); Models include a random intercept and slope for local government area (LGA) and an offset for LGA population; *non-significant interactions were removed from the models
